# Artificial intelligence models in prediction of response to cardiac resynchronization therapy: a systematic review

**DOI:** 10.1007/s10741-023-10357-8

**Published:** 2023-10-20

**Authors:** Wojciech Nazar, Stanisław Szymanowicz, Krzysztof Nazar, Damian Kaufmann, Elżbieta Wabich, Rüdiger Braun-Dullaeus, Ludmiła Daniłowicz-Szymanowicz

**Affiliations:** 1 Faculty of Medicine, Medical University of Gdańsk, Marii Skłodowskiej-Curie 3a, 80-210 Gdańsk, Poland; 2 Visual Geometry Group, University of Oxford, Banbury Road 25, OX2 6NN Oxford, UK; 3 Faculty of Electronics, Telecommunications and Informatics, Gdańsk University of Technology, Gabriela Narutowicza 11/12, 80-233 Gdańsk, Poland; 4 Department of Cardiology and Electrotherapy, Faculty of Medicine, Medical University of Gdańsk, Smoluchowskiego 17, 80-213 Gdańsk, Poland; 5 Department of Cardiology and Angiology, Otto von Guericke University Magdeburg, Leipziger Street 44, 39120 Magdeburg, Germany

**Keywords:** Heart failure, Cardiac resynchronization therapy, Artificial intelligence, Machine learning

## Abstract

The aim of the presented review is to summarize the literature data on the accuracy and clinical applicability of artificial intelligence (AI) models as a valuable alternative to the current guidelines in predicting cardiac resynchronization therapy (CRT) response and phenotyping of patients eligible for CRT implantation. This systematic review was performed according to the PRISMA guidelines. After a search of Scopus, PubMed, Cochrane Library, and Embase databases, 675 records were identified. Twenty supervised (prediction of CRT response) and 9 unsupervised (clustering and phenotyping) AI models were analyzed qualitatively (22 studies, 14,258 patients). Fifty-five percent of AI models were based on retrospective studies. Unsupervised AI models were able to identify clusters of patients with significantly different rates of primary outcome events (death, heart failure event). In comparison to the guideline-based CRT response prediction accuracy of 70%, supervised AI models trained on cohorts with > 100 patients achieved up to 85% accuracy and an AUC of 0.86 in their prediction of response to CRT for echocardiographic and clinical outcomes, respectively. AI models seem to be an accurate and clinically applicable tool in phenotyping of patients eligible for CRT implantation and predicting potential responders. In the future, AI may help to increase CRT response rates to over 80% and improve clinical decision-making and prognosis of the patients, including reduction of mortality rates. However, these findings must be validated in randomized controlled trials.

**Supplementary Information:**

The online version contains supplementary material available at 10.1007/s10741-023-10357-8.

## Introduction

Cardiac resynchronization therapy (CRT) is one of the core treatment methods in chronic heart failure (HF) with reduced left ventricular ejection fraction (LVEF) and a wide QRS complex [1, 2]. According to the 2021 European Society of Cardiology (ESC) guidelines on HF, CRT-Pacemaker/CRT-Defibrillator should be considered for symptomatic patients with HF in sinus rhythm (SR) with a QRS duration (QRSd) ≥ 130 ms (ms) due to left bundle branch block (LBBB) or QRSd ≥ 150 ms if non-LBBB QRS morphology is present and with LVEF ≤ 35% despite optimal medical therapy (OMT) [2]. Clinical outcomes including death or HF hospitalization as well as improvements in the most important echocardiographic parameters, e.g., 6-month reduction in LVESV, have been proposed as outcomes relevant to CRT response [3, 4]. A reduction in morbidity and mortality as well as improvement in cardiac function with an enhancement of quality of life is observed after CRT implantation [1, 2].

Despite relatively clear guidelines, data from the literature report that about 30% of patients who meet eligibility criteria do not respond to CRT treatment [5–11] and identification of the phenotype of an “ideal” CRT responder remains a challenge [2, 12]. Thus, research on accurate prediction of CRT response continues. In addition to electrocardiographic and echocardiographic assessments, advanced computed tomography and magnetic resonance imaging studies are used to predict desired CRT outcomes [13]. Moreover, researchers try to combine several types of data to find new factors predicting positive CRT outcomes [14].

A novel advancement in clinical outcome prediction may result from the use of state-of-the-art statistical modeling, including artificial intelligence (AI) models [15, 16]. It was found that AI can be as good as, or sometimes even better than, health-care professionals in classifying diseases using medical imaging [17]. Moreover, AI can blend, analyze, and interpret a very sophisticated and broad range of different data types that are too complex for human-based analyses [18–23].

Therefore, training of complex AI algorithms seems to be a valuable solution to the task of accurate prediction of CRT response. In comparison with traditional statistics (descriptive statistics, statistical hypothesis testing with the use of *p* value), which describe trends and statistical significance based on the results for a group of patients, supervised AI models (S-AI model) can be used to predict CRT response for a single patient, providing a good basis for a personalized assessment of eligibility for CRT [24]. On the other hand, unsupervised AI models (U-AI model) can identify clusters of patients with homogenous clinical characteristics, including similar CRT outcome, which can reveal the phenotype of a CRT responder [25]. Recently, several studies that aim to utilize U-AI models in phenotyping of CRT respondents as well as S-AI models to predict the outcome of CRT have been published [4, 15, 16, 24–48]. Since this research area develops rapidly and AI has proven to be effective in various clinical settings [17], it is of value to summarize the body of evidence on CRT response prediction with the use of AI.

## Aim

The aim of the presented review is to summarize the literature data regarding the accuracy and clinical applicability of AI models as a valuable alternative to the current guidelines in predicting CRT response and phenotyping of patients eligible for CRT implantation.

For U-AI models, the ability of accurate clustering of CRT (non-)respondents is evaluated. For S-AI models, the accuracy of the patient selection that will benefit from CRT in the future is assessed. Prospective primary outcome events are used as reference standards.

## Methods

### Evidence acquisition

#### Literature search

**Fig. 1:**
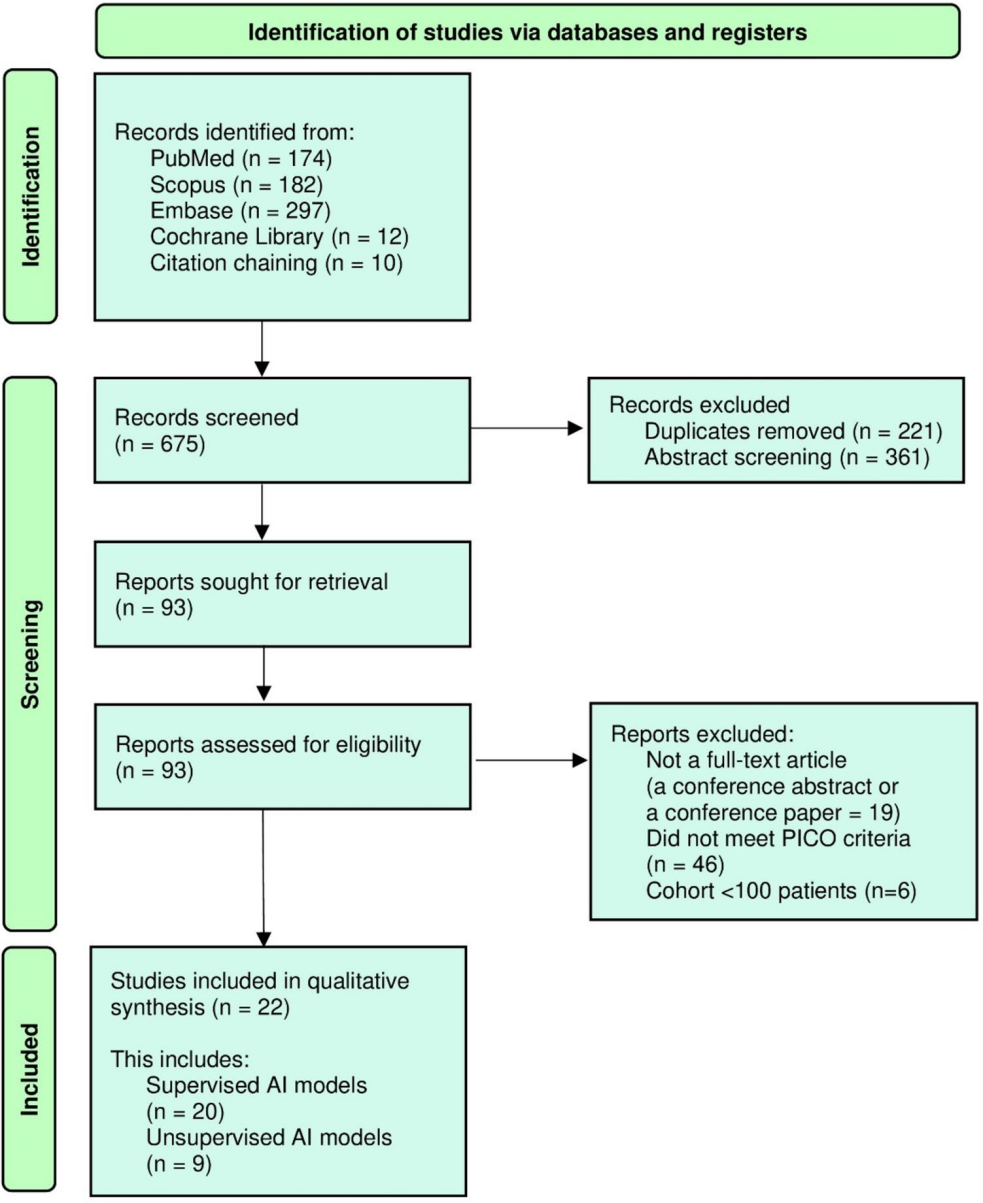
Preferred Reporting Items for Systematic Reviews and Meta-Analyses flowchart. AI—artificial intelligence; PICO—patients, intervention, comparison, outcome

This systematic review was performed in accordance with the Preferred Reporting Items for Systematic Reviews and Meta-Analyses (PRISMA) 2020 statement [49]. Scopus, PubMed, Cochrane Library, and Embase databases were searched for relevant articles (Fig. 1).

Two separate database searches by WN and KN were conducted to identify studies eligible for the systematic review. The last search was performed on 31st May 2023. The basic search query was as follows: “(cardiac resynchronisation therapy OR dyssynchrony) AND (ecg OR electrocardiography OR electrocardiogram OR echocardiography OR cardiac ultrasound OR echocardiogram OR computed tomography OR ct OR magnetic resonanse imaging OR mri OR Single-photon emission computed tomography OR spect) AND (machine learning OR ai OR artificial intelligence OR machine intelligence OR k-means OR random forest OR gradient boost OR support vector machines OR decision tree OR lstm OR long short term network OR encoder OR decoder OR tensorflow OR pytorch OR keras OR classification algorithm OR supervised learning OR unsupervised learning OR clustering OR deep learning OR deep neural network OR cnn OR convolutional neural network OR computer vision OR rnn OR recurrent neural network)”.

The following keywords were chosen as they describe the studied intervention (CRT, group 1), the diagnostic approaches used to qualify the patients for the intervention (electrocardiography and echocardiography, group 2), and the use of AI in the study (group 3). The keywords in the groups were connected with the operator “OR” and the groups of keywords were separated with the operator “AND” to find articles eligible for the review.

Full search strategy for each database is available in Supplementary Material S1.

The preliminary search returned a total of 675 articles. Duplicates were removed. After title and abstract screening, 93 studies were selected for full-text analysis. Articles were excluded due to the following reasons: article did not meet the PICO criteria (46), study description was not a full-text original article (19), and cohort < 100 patients (6). A total of 22 studies were thus included in the review.

#### Selection of AI models

In each study, a maximum of one binary clinical outcome and one binary echocardiographic endpoint of CRT response were identified. Further on, for each binary outcome, a maximum of one supervised and one unsupervised algorithm were identified. Only the best “AI model-primary outcome” pairs were incorporated in the review.

A S-AI model was represented by any machine learning algorithm that was used for supervised classification of patients who will and will not benefit from CRT implantation. An U-AI model was defined as any clustering algorithm that was used for unsupervised phenotyping of CRT responders. Moreover, according to the definition of “artificial intelligence,” the algorithm must have been able to analyze and draw inferences from patterns in data without direct instructions on how to perform it [50]. If an AI model was not described in the article, the study was not included in this review.

For S-AI models, the best AI model for the prediction of a given primary outcome was the AI model with the best overall accuracy score or the AI model chosen and recommended by the authors of the study. For U-AI models, the best AI model was the AI model with the best discrimination power to distinguish between the identified clusters of patients or the AI model chosen and recommended by the authors of the study.

A deep learning algorithm was defined as a prediction model that consists of multiple stacked layers and uses data to capture hierarchical levels of abstraction [51].

#### Data extraction

Only the best AI model-primary outcome analyses were taken into consideration.

The following information was extracted for each analyzed AI model: 1st author, date of publication, objective, study design, number of patients, mean age of patients, percentage of males, percentage of patients with LBBB, percentage of patients with ischemic cardiomyopathy (ICM), inclusion and exclusion criteria, definition of the primary outcome, rate of primary outcome events, type of the best performing algorithm, pre- and post-implantation data used to train the AI model (demographic, clinical, laboratory, medications, echocardiography, electrocardiography, computed tomography, magnetic resonance imaging, ergospirometry, text, genetic, post-lead position, post-intracardiac electrocardiography, post-electrocardiography), number of input features, other algorithms tested, and the method used to handle missing values.

Additionally, for S-AI models, measures of the performance of the best algorithm (accuracy, specificity, sensitivity etc.) and the most predictive variables were extracted. Furthermore, availability of the AI model online was checked. For U-AI models, additional information included the number of clusters identified by the AI model, the results of phenotyping, and the factors associated with CRT response.

If specific information was not obtainable in the original manuscript, it was described as Not Reported (NR). In some cases, the overall efficacy of prediction was reported as the area under the receiver operating characteristic curve (AUC), sometimes as accuracy, sometimes both metrics were available. All of them were always extracted, and each of them was used in an independent statistical analysis.

### PICO question

A PICO-styled research question was formulated to identify studies eligible for the analysis. Respectively, for studies describing AI models based on supervised algorithms:

1. Patients: patients who received CRT-D or CRT-P
2. Intervention (index test): prediction of response to CRT with the use of AI model
3. Comparison: response to CRT defined by the primary outcome (e.g., death, HF hospitalization, LVEF improvement)
4. Outcome: accuracy/AUC of the CRT response prediction capabilities of the AI model

For studies describing AI models based on unsupervised algorithms (clustering and phenotyping of CRT patients), the following PICO was formed:

1. Patients: patients who received CRT-D or CRT-P
2. Intervention: a group of patients with the highest/lowest proportion of CRT responders
3. Comparison: proportion of CRT responders in other group(s) revealed by the phenotyping
4. Outcome: comparison of the proportion of CRT responders between the identified clusters of patients

### Evidence synthesis

All studies meeting the eligibility criteria are summarized in Tables 1 and 2. Additional data are available in the Supplementary Tables S2 and S3. To analyze and compare findings across the studies, counts, percentages, median values, and range were used. Due to large differences in patient inclusion/exclusion criteria as well as various primary outcome definitions across the reviewed studies, a quantitative synthesis of the results (meta-analysis) was not possible.

**Table 1:** Unsupervised artificial intelligence models

Author and date	Design	*N*	Definition of the primary outcome	Best algorithm	*N* of clusters	Results
Bivona et al. (2022)	Retrospective cohort study	200	Death (median follow-up of 4 years)	Gaussian mixture model	3	3 response clusters: 10%, 40%, and 66% deaths (overall death rate: 26%); cluster 2 vs cluster 1: HR 1.84 (95% CI 1.35–2.50; *p* < 0.001); cluster 3 vs cluster 1: HR 2.23 (95% CI 1.71–2.90; *p* < 0.001)
Cikes et al. (2018)	RCT	1106	Death from any cause or a non-fatal HF event (average follow-up of 2.3 years)	Multiple kernel learning and k-means clustering	4	Phenogroups 1 and 3 were associated with a substantially better treatment effect of CRT-D on the primary outcome (HR 0.36; 95% CI 0.19–0.68; *p* < 0.001 and HR 0.35; 95% CI 0.19–0.64; *p* < 0.001) than observed in the other groups
Fenny et al. (2020)	Retrospective cohort study	840	Composite of death, heart transplant, placement of left ventricular assist device at 12-month follow-up	k-means clustering	2	Compared with group 2, group 1 had lower risk for reaching the composite end point (HR 0.44 [95% CI 0.38–0.53]; *p* < 0.001)
Gallard et al. (2021)	Prospective cohort study	250	Decrease in LVESV of ≥ 15% at 6-month follow-up	k-means clustering	5	Five clusters were identified, with response rates of 50%, 71%, 72%, 86%, and 93%, respectively (overall response rate was 74%)
Gallard et al. (2021)	Prospective cohort study	250	Composite of death and hospitalization for HF (mean duration of follow-up was 3.7 years)	k-means clustering	5	Five clusters were identified, with adverse event rates of 37%, 14%, 14%, 14%, and 7%
Galli et al. (2021)	Prospective cohort study	193	Composite of heart transplantation, LV assist device implantation, or all-cause death (median follow-up of 37 months)	k-medoid clustering	2	In comparison to group 1, group 2 had higher outcome reach: HR 4.70 (95% CI 2.1–10.0)
Galli et al. (2021)	Prospective cohort study	193	Decrease in LVESV of ≥ 15% at 6-month follow-up	k-medoid clustering	2	Groups 1 and 2 had 89% and 34% responders, respectively (overall response rate: 68%)
Riolet et al. (2021)	Retrospective cohort study	328	Death from any cause (median follow-up of 51 months)	Agglomerative hierarchical clustering based on k-means	4	In comparison to group 1, groups 2, 3, and 4 had greater risk of death: HR 0.89 (95% CI 0.47–1.70), HR 3.23 (95% Cl 1.9–5.5), and HR 2.49 (95% CI 1.38–4.50)
Riolet et al. (2021)	Retrospective cohort study	328	Decrease in LV end-systolic volume of ≥ 15% at 9-month follow-up	Agglomerative hierarchical clustering based on k-means	4	In groups 1 to 4, the response rates were 81%, 78%, 39%, and 59%, respectively

*HF* heart failure, *LV* left ventricle, *LVESV* left ventricle end-systolic volume, *RCT* randomized controlled trial

**Table 2:** Supervised artificial intelligence models

Author and date	Design	*N*	Definition of the primary outcome	Best algorithm	Performance of the algorithm	Validation strategy (training/validation proportion)	Availability
Bivona et al. (2022)	Retrospective cohort study	200	Death (median follow-up of 4 years)	Logistic regression	AUC 0.86	Internal fivefold cross-validation	http://gmmxcrt.pythonanywhere.com
Cai et al. (2021)	Retrospective cohort study	1664	Absolute improvement of > 5% in LVEF measured at 6‐month follow‐up	Stacked contractive autoencoder and ensemble of bagging/adaboost/xgboost	AUC 0.76	Internal fivefold cross-validation (70%), internal hold-out test set (30%)	NR
Feeny et al. (2019)	Retrospective cohort study	925	10% absolute LVEF increase at 12-month follow-up	Naive Bayes classifier	AUC 0.70	Internal fivefold cross-validation	http://riskcalc.org:3838/CRTResponseScore
Fernandes et al. (2023)	Prospective cohort study	158	LVEF improvement of ≥ 5% at 6-month follow-up	Prediction analysis of microarrays	AUC 0.80, sensitivity 0.86, specificity 0.75	Internal threefold cross-validation with 25 repeats, internal hold-out test set (20%)	NR
Field et al. (2020)	RCT	419	Decrease in LVESV ≥ 15% at 6-month follow-up	Classification tree	Probability of response in range from 30 to 63%	Internal cross-validation	Yes, in the publication
Gallard et al. (2020)	Retrospective cohort study	323	Decrease in LVESV ≥ 15% at the 6-month follow-up	Random forest with feature selection	AUC 0.81	Internal Monte Carlo cross-validation (80%/20%)	NR
Galli et al. (2021)	Prospective cohort study	193	Decrease in LVESV ≥ 15% at 6-month follow-up	Ensemble learning: Boruta algorithm and random forest	AUC 0.81	Internal validation	NR
Galli et al. (2021)	Prospective cohort study	193	Composite of heart transplantation, LV assist device implantation, or all-cause death (median follow-up of 37 months)	Ensemble learning: Boruta algorithm and random forest	AUC 0.84	Internal validation	NR
Haque et al. (2022)	RCT	794	Decrease in ESV ≥ 15 mL at 6 months post-implantation	Ensemble of nine equally weighted algorithms	AUC 0.78	Internal fivefold cross-validation (45%), internal hold-out test set (20%)	https://github.com/SysMechBioLab/CRT_IML
He et al. (2023)	Retrospective cohort study	130	LVEF improvement of ≥ 5% at 6-month follow-up	Ensemble of unsupervised neural network and logistic regression	AUC 0.74	External validation	NR
Hong et al. (2022)	Prospective cohort study	280	Adverse outcome (-) [death, heart transplantation, extracorporeal membrane oxygenation (ECMO), or use of a ventricular assist device] OR LVEF increase at 12-month follow-up or earlier	Classification tree	–	Internal validation	Yes, in the publication
Howell et al. (2021)	RCT	741	Freedom from death, HF hospitalization, and a decrease in LVESV ≥ 15% at 6-month follow-up	Adaptive lasso model	AUC 0.76	Internal tenfold cross-validation (80%), internal hold-out test set (20%)	https://ecgpredictscd.org/crt
Hu et al. (2019)	Retrospective cohort study	990	Composite of < 0% improvement in LVEF 6–18 months post-procedure or death by 18 months	Ensemble learning: machine learning and natural language processing	AUC 0.75, 65% accuracy, 79% specificity, 26% sensitivity	Internal fivefold cross-validation (80%), internal hold-out test set (20%)	NR
Kalscheur et al. (2018)	RCT	1076	Death or heart failure hospitalization at 12-month follow-up	Random forest	AUC 0.74, 52% sensitivity, 38% NPV, 80% specificity, 88% PPV	Internal tenfold cross-validation (45%), internal hold-out test set (55%)	NR
Lei et al. (2019)	Retrospective cohort study	117	Improvement in NYHA functional ≥ 1 and a decrease in LVESV ≥ 15% at 6-month follow-up	Support vector machines	85% accuracy	Internal tenfold cross-validation	NR
Liang et al. (2021)	Retrospective cohort study	752	> 10% absolute LVEF increase at 12-month follow-up	Ridge regression	AUC 0.77, 92% specificity, 54% sensitivity	Internal tenfold cross-validation	http://www.crt-response.com
Schmitz et al. (2014)	Case–control study	156	Decrease in LVESV ≥ 15% at 6- to 12-month follow-up (median 9 months)	PART algorithm	85% accuracy, 88% specificity, 82% sensitivity	Internal tenfold cross-validation	Yes, in the publication
Tokodi et al. (2020)	Retrospective cohort study	1510	Death at 5-year follow-up	Random forest	AUC 0.80	Internal tenfold cross-validation	https://arguscognitive.com/crt
Wouters et al. (2023)	Retrospective cohort study	1306	Left ventricular assist device (LVAD) implantation, heart transplantation (HTx), and all-cause mortality (median follow-up 3.5 years)	FactorECG (neural network consisting from variational auto-encoder and decoder)	AUC 0.69	Internal validation by means of boostrap	https://crt.ecgx.ai
Wouters et al. (2023)	Retrospective cohort study	821	Decrease in LVESV ≥ 15% at 6 to 12-month follow-up	FactorECG (neural network consisting from variational auto-encoder and decoder)	AUC 0.69	Internal validation by means of boostrap	https://crt.ecgx.ai

*AUC* area under the receiver operating characteristic curve, *HF* heart failure, *LV* left ventricle, *LVEF* left ventricle ejection fraction, *LVESV* left ventricle end-systolic volume, *NPV* negative predictive value, *RCT* randomized controlled trial, *PPV* positive predictive value

### Quality assessment

The risk of bias (ROB) of included studies was performed by the first author of the study (WN). It was assessed according to the Prediction model Risk Of Bias ASsessment Tool (PROBAST) [52].

ROB was assessed in the following 4 domains: participants, predictors, outcome, and analysis. A total of 20 signaling questions were used to facilitate a structured assessment of the ROB, which was defined as occurring when shortcomings in study design, conduct, or analysis lead to systematically distorted estimates of a given model’s predictive performance. The first three domains were also used for applicability assessment. Last but not least, an overall assessment of the ROB and applicability of the prediction model was completed. A “ + ” indicates low ROB/low concern regarding applicability; “ − ” indicates high ROB/high concern regarding applicability; and “?” indicates unclear ROB/unclear concern regarding applicability [52]. For each eligible study, a total of 9 analyses (+ / − /? assessments) were conducted. In this review, if any domain of ROB or applicability assessment was rated as high/unclear ROB, the “overall ROB”/ “overall applicability” domains were also rated as high or unclear ROB. Since S-AI models aim at the prediction of response to CRT, only these AI models were analyzed with the use of the PROBAST. A detailed table is available in the Supplementary Material S1.

## Results

### General characteristics

This review included a total of 22 studies, which reported 29 separate AI model-primary outcome analyses with a total of 14,258 patients (Fig. 1). There were 20 S-AI models and 9 U-AI models (*N* = 11,743 and *N* = 2917 patients, respectively). These were further divided into echocardiography outcome-based and clinical outcome-based U-AI models as well as into echocardiography outcome-based and clinical outcome-based S-AI models (Tables 1, 2, and 3, Supplementary Tables S2, S3, and S4). The majority of studies included in our analysis have been published during the last three years.

**Table 3:** General characteristics of the analyzed artificial intelligence models

Characteristics	All models	Unsupervised models	Unsupervised models (ECHO outcome)	Unsupervised models (clinical outcome)	Supervised models	Supervised models (ECHO outcome)	Supervised models (clinical outcome)
**General characteristics of all models**							
***N* of AIMs (Nof studies)**	29 (22)	9 (6)	3 (3)	6 (6)	20 (18)	11 (11)	9 (9)
***N* of patients included in all studies**	14,258	2917	771	2917	11,743	6335	6413
**General characteristics per each model**							
**Date of publication, median (range)**	2021 (2014–2023)	2021 (2018–2022)	2021 (2021–2021)	2021 (2018–2022)	2021 (2014–2023)	2021 (2014–2023)	2021 (2018–2023)
***N* of patients, median (range)**	328 (117–1664)	250 (193–1106)	250 (193–328)	289 (193–1106)	580 (117–1664)	419 (130–1664)	741 (117–1510)
**Age (mean number of years), median (range)**	67 (60–72)	67 (64–72)	67 (67–72)	67 (64–72)	66 (60–72)	66 (60–69)	67 (65–72)
**Males in %, median (range)**	68 (50–87)	67 (50–75)	66 (50–71)	69 (50–75)	69 (58–87)	69 (63–87)	68 (58–76)
**LBBB ( +) in %, median (range)**	75 (9–100)	75 (63–88)	87 (72–88)	73 (63–88)	74 (9–100)	77 (42–100)	72 (9–88)
**ICM ( +) in %, median (range)**	43 (9–59)	37 (31–56)	33 (31–37)	40 (31–56)	48 (9–59)	48 (9–59)	46 (19–57)
**Primary outcome event rate (%), median (range)**	47 (15–78)	32 (15–78)	68 (65–74)	22 (15–78)	48 (15–73)	51 (42–69)	32 (15–73)
**Number of groups, median (range)**	2 (2–5)	4 (2–5)	4 (2–5)	3 (2–5)	2 (2–2)	2 (2–2)	2 (2–2)
**Accuracy of the best model, median (range)**	–	–	–	–	78 (63–85)	78 (63–85)	75 (65–85)
**AUC of the best model, median (range)**	–	–	–	–	0.76 (0.69–0.86)	0.76 (0.69–0.81)	0.75 (0.69–0.86)
**Models achieving ≥ 70% accuracy or AUC ≥ 0.70, *N* (%)**	–	–	–	–	16 (80)	9 (82)	7 (78)
**Models achieving ≥ 80% accuracy or AUC ≥ 0.80, *N* (%)**	–	–	–	–	8 (40)	4 (36)	4 (44)
***N* of input features, median (range)**	20 (2–487)	50 (3–70)	55 (28–70)	39 (3–70)	16 (2–487)	16 (2–487)	15 (3–45)
***N* of types of input data, median (range)**	5 (1–7)	5 (1–7)	5 (5–7)	5 (1–7)	5 (1–7)	5 (1–7)	5 (1–7)
***N* of all algorithms tested, median (range)**	1 (1–15)	1 (1–2)	1 (1–1)	1 (1–2)	2 (1–15)	5 (1–15)	1 (1–8)

*AHA* American Heart Association, *AI* artificial intelligence, *AUC* area under the receiver operating characteristic curve, *ESC* European Society of Cardiology, *ECHO* echocardiographic, *HF* heart failure, *LVEF* left ventricle ejection fraction, *LVESV* left ventricle end-systolic volume, *NYHA* New York Heart Association, *N* number, *%* percentage

Most of the AI models were based on data collected in retrospective cohort studies (*N* = 16, 55%), followed by prospective cohort studies (*N* = 8, 28%), randomized controlled trials (RCTs, *N* = 5, 17%), and case–control studies (*N* = 1, 3%). Each AI model was trained and validated on a median number of 328 patients (range 117–1668) with a median age of 67 years (range 60–72). The median rate of primary outcome events was 47% (range 15–78%). The median percentage of male patients across the analyzed AI models was 68% (range 50–87%).

The most frequent inclusion criteria included QRSd ≥ 120 ms, NYHA class, and reduced LVEF ≤ 35%. The most frequently reported primary endpoints included death, a decrease in LV end-systolic volume (LVESV), a LVEF improvement, and an occurrence of an HF event. Thirty-three percent of the AI models were based on a composite outcome.

The majority of AI models were trained with a non-deep learning algorithm. The median number of types of input data the AI models were trained on was 5 (range 1–7), and input features, 20 (range 2–487). The most often used types of input features were pre-implantation features such as echocardiographic parameters, followed by clinical data, demographic characteristics, and electrocardiographic data. Most of the AI models did not have any post-implantation input data.

### Unsupervised AI models

A total of 3 echocardiographic outcome-based unsupervised AI models (EU-AI models) and 6 clinical outcome-based unsupervised AI models (CU-AI models) were included in the review (771/2917 patients, respectively; Tables 1, and 3, Supplementary Tables S2 and S4). The median number of patients for EU-AI models was 250 (range 193–328) and 289 (range 193–1106) for CU-AI models. The median rate of primary outcome events was 68% (range 65–74%) and 22% (range 15–78%), respectively. The median number of clusters identified by EU-AI models was equal to 4 (range 2–5) and 3 (range 2–5) for CU-AI models. The most used input data for U-AI models were echocardiographic, clinical, laboratory, and demographic characteristics. Almost all U-AI models came from retrospective and prospective cohort studies. The median number of input features used to train an EU-AI model was equal to 55 (range 28–70) and 39 (range 3–70) to train a CU-AI model. The median number of input data types, 5, was the same for EU- and CU-AI models.

Most commonly, the patients were qualified for CRT implantation based on wide QRSd of ≥ 120 ms, NYHA class, and reduced LVEF of ≤ 35%. The most frequently reported primary outcome measures were death, HF event, and a decrease in LVESV. Forty-four percent of the AI models had a composite primary endpoint. The clustering of CRT patients was mostly based on k-means clustering or k-medoid algorithms. Independent of the primary outcome as well as the number of clusters identified by the U-AI models, statistically significant differences in the rate of primary outcome events across the observed clusters were observed in each study.

**Fig. 2:**
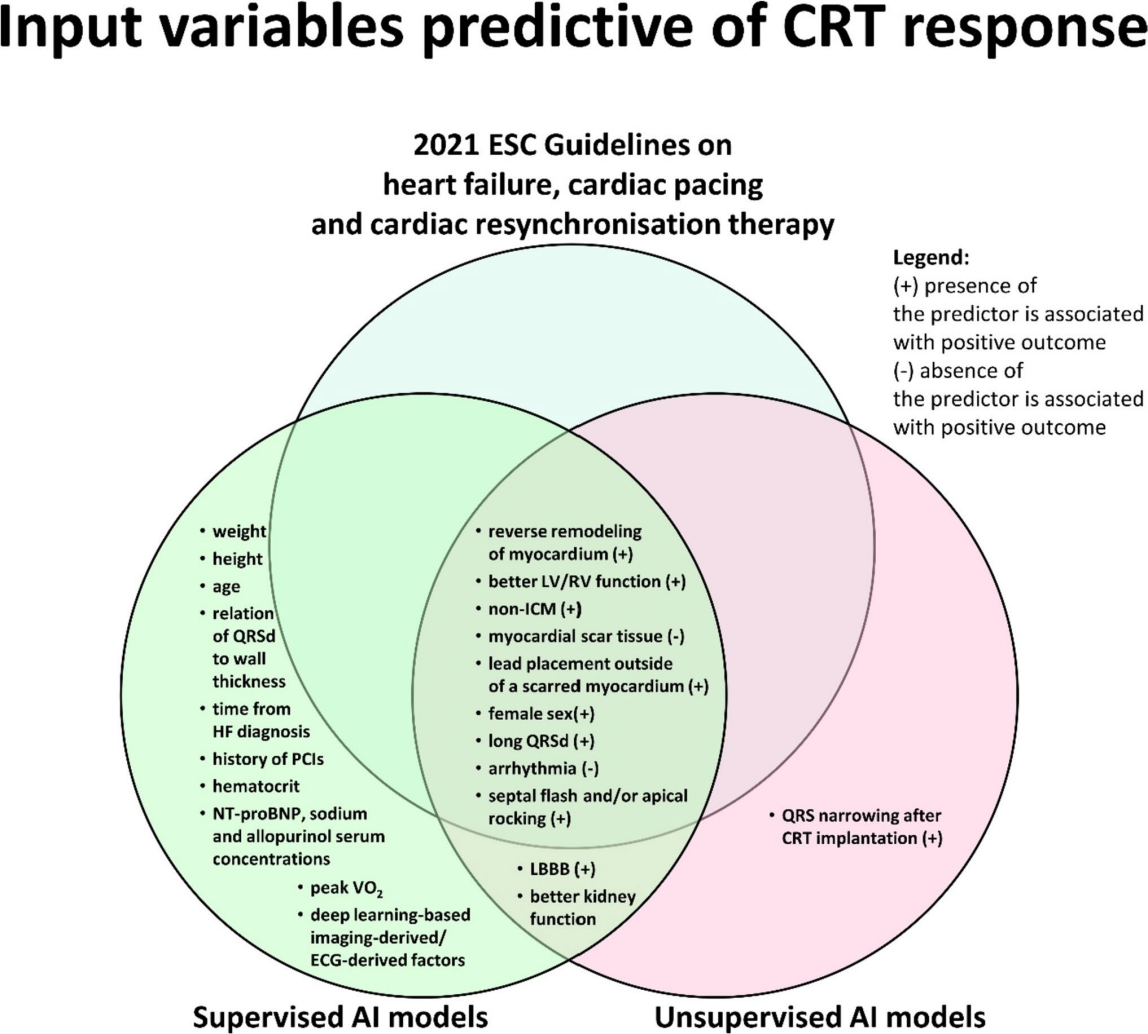
Input variables predictive of CRT response. AI—artificial intelligence; CRT—cardiac resynchronization therapy; ESC—European Society of Cardiology; ECG—electrocardiography; HF—heart failure; LBBB—left bundle branch block; LV—left ventricle; non-ICM—non-ischemic cardiomyopathy; NT-proBNP—N-terminal pro–brain-type natriuretic peptide; PCI—percutaneous coronary intervention; peak VO_2_—maximal oxygen consumption; QRSd—QRS duration; RV—right ventricle

Various clinical, echocardiographic, and electrocardiographic variables were associated with more favorable outcomes (Fig. 2). However, in one study, LVEF and QRSd were not related to more positive outcomes.

### Supervised AI models

A total of 11 echocardiographic outcome-based supervised AI models (ES-AI models) and 9 clinical outcome-based supervised AI models (CS-AI models) were included in our review (6335/6413 patients, respectively; Tables 2, and 3, Supplementary Tables S3 and S4). The median number of patients for ES-AI models was 419 (range 130–1664) and 741 (range 117–1510) for CS-AI models. The median primary outcome reach was 51% (range 42–69%) and 32% (range 15–73%), respectively. In all described studies, always two groups of patients (responders/non-responders) were identified.

The median accuracy of ES-AI models and CS-AI models was equal to 78% (range 63–85%) and 75% (65–85%), respectively. The ES-AI models and CS-AI models achieved median AUCs of 0.76 (range 0.69–0.81) and 0.75 (range 0.69–0.86), respectively. Schmitz et al. created the best ES-AI model (85% accuracy, PART algorithm). In the group of CS-AI models, Bivona et al. proposed the best algorithm (AUC of 0.86, logistic regression algorithm). As many as 80% of all S-AI models achieved ≥ 70% accuracy/AUC ≥ 0.70 and 40% of S-AI models achieved ≥ 80% accuracy/AUC of ≥ 0.80.

Echocardiographic, clinical, demographic, and electrocardiographic characteristics made for the most commonly used input data for S-AI models. The majority of the S-AI models were based on data collected in retrospective cohort studies, followed by prospective cohort studies and RCTs. The median number of input features used to train an ES-AI model was equal to 16 (range 2–487) and 15 (range 3–45) to train a CS-AI model. The median number of input data types, 5, was the same for ES- and CS-AI models.

Inclusion criteria varied across the analyzed studies, but most of them qualified patients based on wide QRSd of ≥ 120 ms, NYHA class, and reduced LVEF of ≤ 35%. A decrease in LVESV and an absolute LVEF increase as well as death were the most frequently reported primary outcome measures, while 31% of the models had a composite outcome. Majority of algorithms used to create CRT prediction models were non-deep learning algorithms (85%), e.g., support vector machines or random forest algorithms.

Similar to the U-AI models, in S-AI models various clinical, echocardiographic, electrocardiographic, and laboratory characteristics were identified as the most predictive variables in the classification of CRT responders and non-responders (Fig. 2).

Almost all algorithms were validated internally, most commonly with the use of the tenfold or fivefold cross-validation protocol. Six studies (30%) were evaluated using an additional internal hold-out test dataset.

A total of 11 S-AI models are available online. The most accurate publicly available ES-AI model and CS-AI model were reported by Schmitz et al. (85% accuracy, > 100 patients) and Bivona et al. (AUC of 0.86, based on > 100 patients), respectively.

**Fig. 3:**
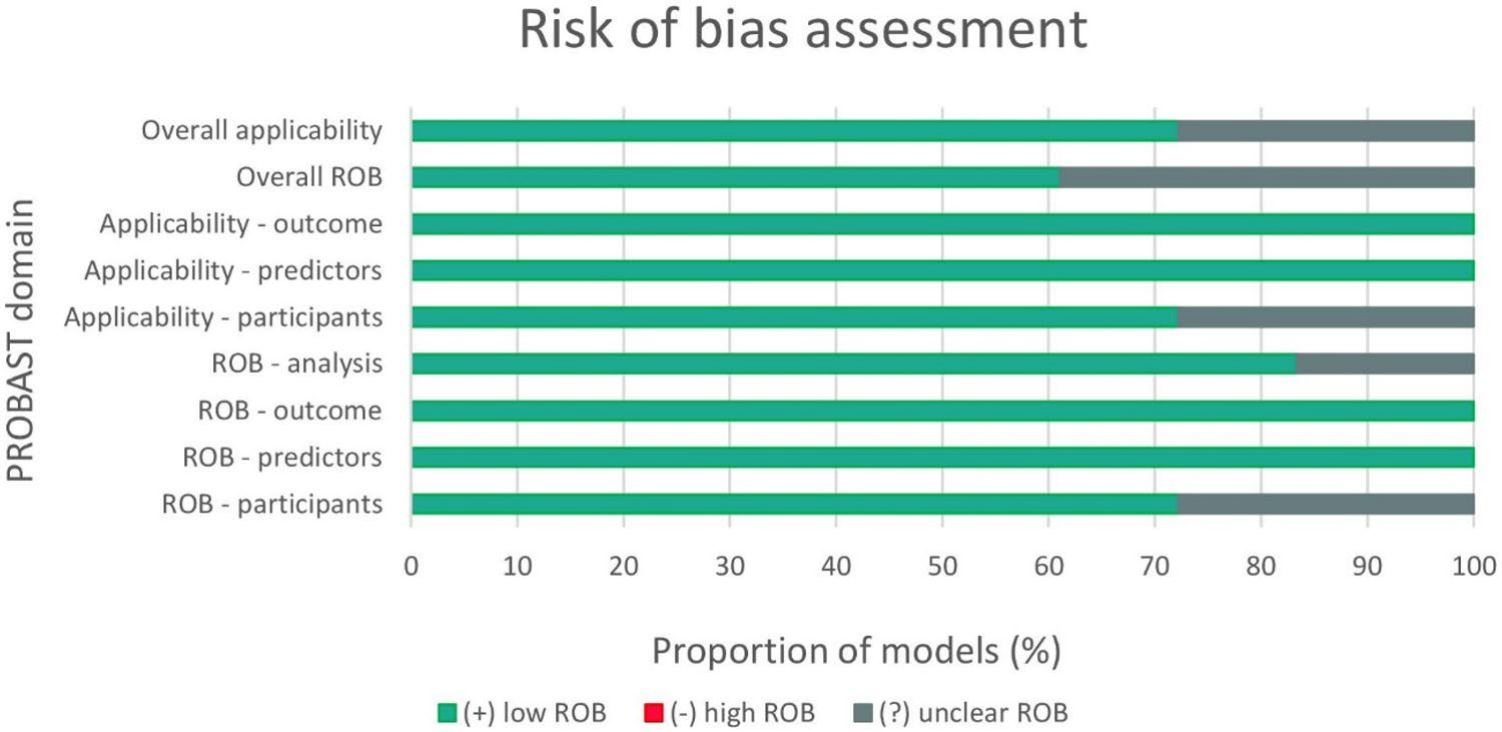
Risk of bias assessment with the use of the PROBAST. AI—artificial intelligence; ROB—risk of bias

A total of 11 S-AI models (61%) had low overall ROB whereas 13 S-AI models (72%) had a low overall concern regarding applicability (Fig. 3). Unclear ROB in the domain “ROB participants” and “applicability participants” was allocated due to an unclear description of inclusion/exclusion criteria. Three S-AI models were rated as “unclear ROB” in the domain “ROB analysis” due to the absence of an explanation of how missing data were handled.

## Discussion

### General characteristics

**Fig. 4:**
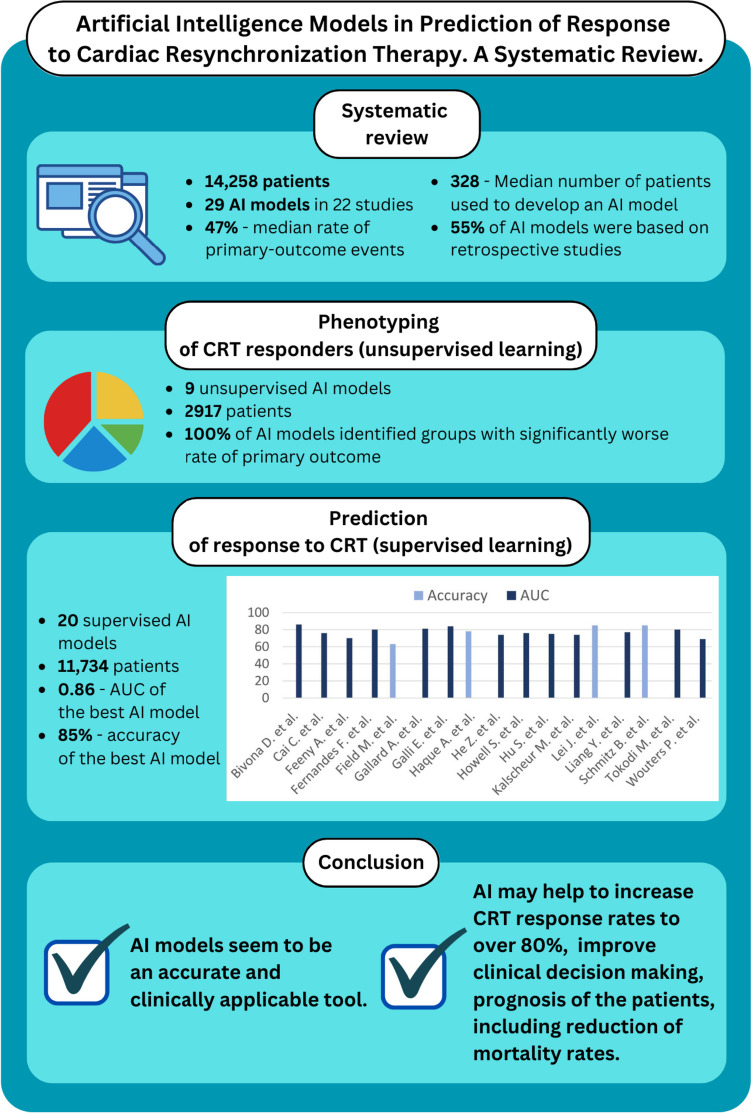
Summary of key characteristics of analyzed artificial intelligence models. AI—artificial intelligence; AUC—area under the receiver operating characteristic curve; CRT—cardiac resynchronization therapy; QRSd—QRS duration; NYHA—New York Heart Association; HF—heart failure; LVEF—left ventricle ejection fraction; LVESV—left ventricle end-systolic volume

To the authors’ knowledge, this is the first comprehensive systematic review on the use of AI algorithms in CRT response prediction and phenotyping of patients qualified for CRT. Summarizing a total of 22 studies, involving 14,458 individuals, and describing 20 S-AI models as well as 9 U-AI models, this systematic review can provide a solid foundation for future research on the use of AI in CRT (Fig. 4).

Most of the AI models described in this review included patients based on QRSd ≥ 120 ms, NYHA class, reduced LVEF ≤ 35%, and the condition of having an OMT prior to CRT implantation, which tends to be in line with the most recently published guidelines [1, 2]. The percentage of AI models based on retrospective and prospective studies was 55% and 45%, respectively. The populations included in this review were moderately homogenous, with the median age of 67 years old (range 60–72), the median proportion of male patients across the analyzed AI models of 68% (range 50–87%), and a wide variety of cohort sizes on which the AI models were trained (median number of patients = 328, range 117–1668). In addition, the use of a wide variety of echocardiographic and clinical outcomes that were based on different follow-up intervals resulted in a heterogenous rate of primary outcome events (median = 47%, range 15–78%).

### Unsupervised AI models

Three EU-AI models and six CU-AI models met the eligibility criteria for this review. The patient inclusion criteria were homogenous (QRSd ≥ 120 ms, NYHA, reduced LVEF ≤ 35%, OMT), as most of the studies were based on the official guidelines [53]. Interestingly, even though a wide range of clusters of patients across the reviewed U-AI models was identified (median *N* of clusters = 4, range 2–5), which were based on various numbers of input features (median = 50, range 3–70), different numbers of patients were used to train the AI models (median = 250, range 193–1106) and a divergent rate of primary outcome events, especially for CU-AI models (median = 22%, range 15–78%), each of the U-AI models was able to separate clusters of patients with a significantly better prognosis than the rest of identified groups of patients. Therefore, it seems that nowadays available U-AI models can accurately extract phenotypes of patients with worse response to CRT independently of the input data.

### Supervised AI models

Eighteen ES-AI models and nine CS-AI models were included in the review. Only about 80% of the analyzed studies included a direct description of the key guideline-recommended CRT inclusion criteria [1, 2, 53] (wide QRSd, NYHA class, reduced LVEF), increasing the potential ROB and rising applicability concerns. Similar to the U-AI models, even though the S-AI models were based on various types of input variables (median = 5, range 1–7), different numbers of input features (median = 16, range 2–487) and cohort sizes (median = 580, 130–1664) had a large discrepancy primary outcome event rate (median = 48%, range 15–73%), as many as 80% of them had ≥ 70% accuracy/AUC of ≥ 0.70, and 40% of them were able to achieve ≥ 80% accuracy/AUC of ≥ 0.80 in the prediction of response to CRT. Schmitz et al. [39] created the best ES-AI model (85% accuracy), while Bivona et al. [26] proposed the best CS-AI model (AUC of 0.86). Both algorithms are publicly available.

In two studies, a large number of input features (> 100) were used. Such a large number of input features were obtained by combining demographic, clinical, laboratory, medication, echocardiographic, and electrocardiographic data (487 features in total) [4]. On the other hand, Gallard et al. derived additional input data (311 features in total) with the use of advanced mathematical computations. However, in the end, only the 14 most important characteristics were selected to train the final model [16].

### Clinical adoption of supervised AI models

To be clinically applicable, an S-AI model should require simple, routinely collected input data and have high performance. Moreover, the algorithm should be trained on a large, prospectively observed cohort and predict a valuable primary endpoint.

Schmitz et al. [39] and Bivona et al. [26] proposed AI models with the best performance (85% accuracy and 0.86 AUC, respectively). Increasing the CRT prediction accuracy from recommendation-based 70% to AI-based 85% would halve the number of potential non-responders and lead to a significantly higher clinical and economic effectiveness of CRT. However, these AI models are based on highly sophisticated mathematical computations and genetic analyses and require not routinely measured pre- and post-implantation data (e.g., peak VO_2_ before and 6 months after CRT implantation).

Relatively high-performing AI models trained on routinely collected data (demographic, clinical, echocardiographic, and electrocardiographic parameters) were proposed by Galli et al. [30] (AUC of 0.84 and 0.81), Tokodi et al. [41] (AUC of 0.80), and Howell et al. [31] (AUC of 0.76), but only the last two are publicly available. Moreover, presented S-AI models performed significantly better in CRT response classification than the official guidelines or many other previously created CRT response scores that were not based on AI algorithms [31, 35, 41, 43].

Cai et al. have proven that the increase in input data types improves the performance [4]. However, the relationships are not linear and hard to predict. For example, when guideline-based parameters are enriched with medication, echocardiographic, and demographic data, the AUC increases from 0.56 to 0.69, but further addition of laboratory parameters boosts the AUC to just 0.70 (+ 0.01). On the contrary, when clinical as well as experimental ECG parameters are added, the AUC increases from 0.70 to 0.73 and 0.76, respectively.

Interestingly, Wouters et al. propose a high-performance single-modality ECG-based model [43] (AUC 0.69, trained on 1306 patients). Performing better than the official guideline criteria and requiring just one modality of routinely collected data, it shows a promising perspective for a clinical implementation of models that are both time-effective in terms of data collection and accurate when it comes to CRT response prediction. The model is publicly available [43]. Moreover, when additional clinical data were added to the raw ECG model, the authors notice an increase in performance of only 4.3% (from 0.69 to 0.72). Therefore, this model sacrifices a small decrease in performance for a large boost in potential clinical applicability and proves the concept of “simple input and high-performance AI”.

### The role of ECG in AI-based predictions

According to the most recent guidelines, wide QRSd is a primary characteristic associated with higher response rates after CRT implantation [1]. Pre- and post-implantation electrocardiographic data were used in 59% (*n* = 17) and 6% (*n* = 2) of AI models, respectively. So far, the most clinically applicable supervised AI model in CRT response prediction is the one proposed by Wouters et al. (AUC 0.69, publicly available) [43]. It utilizes raw 12-lead ECG signal processing with the use of deep learning. Interestingly, raw ECG signal can also be used in unsupervised phenotyping of patients and accurately classify groups of patients with significantly different survival probabilities [25]. Thus, AI is able to extract and process raw information without preprocessing.

Nevertheless, in the vast majority of cases, preprocessed data of proven clinical relevance (presence of LBBB, QRSd, QTc duration, etc.) were used as input data. They were used by the most accurate algorithms and were proved to be among the most relevant features in CRT response prediction (Tokodi et al. AUC 0.80 [41], Cai et al. AUC 0.76 [4]).

### Challenging subgroups of CRT recipients

It is well known that one of the most challenging phenotypes of commonly qualified patients is a male with non-LBBB, ICM, and QRSd < 150 ms [1]. To date, there is no study that describes a supervised AI model (CRT response prediction) for the “challenging” subgroups. However, supervised algorithms revealed previously mentioned characteristics to be negatively correlated with CRT response [30, 41, 44]. Moreover, unsupervised algorithms demonstrated that groups of patients associated with this phenotype have significantly worse survival and/or CRT response rates [15, 30, 38]. A detailed description of features predictive for CRT outcome is available in the next section.

### Features predictive for CRT outcome

According to the 2021 ESC guidelines, symptomatic patients with HF in SR, wide QRSd, and reduced LVEF ≤ 35% despite OMT are eligible for a CRT [1, 2]. Moreover, several additional factors including more extensive reverse remodeling of the myocardium, non-ischemic etiology of HF, absence of myocardial scar tissue, female sex, longer QRSd, presence of septal flash, and/or apical rocking in an echocardiographic study as well as lead placement outside of a scarred myocardium are predictive for a favorable CRT response [1, 2].

Based on the identified clusters of CRT patients, most commonly the presence of LBBB, longer QRSd, non-ischemic cardiomyopathy, and smaller dilatation as well as better LV and RV functions were associated with more favorable outcomes (Fig. 2). In addition to that, many clinical, demographic, electrocardiographic, and echocardiographic characteristics identified by the supervised AI models were predictive for CRT response. This emphasizes the need for the adoption of a holistic approach to the problem of a CRT responder selection, which should include an evaluation of a wide range of cardiovascular disease risk factors. Moreover, identification of the same predictors of (non-)response to CRT by the AI models that are endorsed by the most recent guidelines validates the robustness and credibility of the use of AI in this field (Fig. 2).

### Future directions

Since many AI algorithms can predict CRT response with over 80% accuracy, while guideline-based CRT predictions report about 70% of overall accuracy, it would be beneficial to conduct an RCT to prove the potential superiority of AI algorithms over the official guidelines. Without robust, prospective validation in a real-world clinical setting, algorithm’s safety and readiness for implementation in patient care remain unknown [19]. A double-blind two-arm RCT is recommended (control group: patients are selected according the recent guidelines on CRT; experimental group: patients are selected according the classification outcome of the AI-model; primary endpoint: (non-)response to CRT defined by death/HF hospitalization/no improvement in LVEF) [54]. Such approach minimizes potential bias and rigorously examines cause-effect relationships between an intervention and outcome [54].

There is also some indication that AI models can achieve over 90% accuracy [27, 36], but the studies so far have been based on small groups of patients and the reliability of the proposed S-AI models must be validated on larger cohorts. In addition to that, only three AI models used deep learning for CRT response prediction while all the other AI models were based on non-deep learning AI algorithms. As deep learning has changed the landscape of many challenges in computer vision, e.g., image classification [55], it is possible that such methods could result in a boost in performance in the field of CRT response prediction, too. Moreover, many of the best-performing algorithms are based on highly sophisticated imaging techniques generating a lot of data that are not routinely performed and collected for each patient [26, 39].

In general, electrocardiography is considered weaker in terms of clinical relevance which influenced the class of this recommendation in the latest guidelines [1]. Interestingly, although raw electrocardiographic signal was rarely analyzed using machine learning, it shows considerable promise in CRT response prediction [25].

Research on highly accurate (≥ 90%) AI models trained on a large number of input data is highly desirable. This will lead to more robust models that will have higher chances of being generalizable to other cohorts. On the other hand, research on models requiring a small amount of routinely collected data (demographic, clinical, electrocardiographic, and echocardiographic) is also needed. It would be probably less accurate than the more advanced models based on, for example, genetic information [39], but it would be useful in preliminary screening for patients probably eligible for CRT and for qualification for additional diagnostic procedures required by more sophisticated AI models to make the final decision on CRT implantation. Moreover, just 11 out of 20 analyzed S-AI models were shared publicly and free of charge. Thus, the accessibility of AI models should be improved, too.

According to the current state of knowledge and data provided by the literature, the importance of many clinical characteristics (for example, post-implantation lead location, type of input data used) that could theoretically improve the accuracy of CRT response prediction remains unclear and requires future investigation. Apart from the AI algorithm training and validation, the algorithm’s clinical applicability and performance depend on many aspects like inclusion/exclusion criteria or cohort size. Thus, it remains a complex and yet unsolved matter.

Finally, due to the large variety of reviewed studies (various inclusion/exclusion criteria, outcome measures, size of cohorts, types of AI models used, etc.) as well as inconsistency in the reporting, it is not possible to provide any robust recommendations for future AI research in CRT response prediction. Reviewed studies lay the cornerstone for the high-performance, data-driven medicine of the future. We are looking forward to the synergy of machine-aided data analysis and human-driven interpretation of the outcomes.

### Limitations

It is worth mentioning that information about the guideline-recommended inclusion criteria [1, 2] (wide QRSd, NYHA class II–IV, reduced LVEF, OMT) was not available for about 20 to 30% of analyzed AI models. For example, a few authors reported that patients eligible for the development of an AI model were qualified based just on “CRT implantation” [4, 24, 40, 41] or “CRT implantation and QRSd ≥ 120 ms” [35] which is a very broad term, encompassing not only patients with dyssynchrony but also other conditions such as sinus node dysfunction or atrioventricular blocks [1]. This issue reduced the quality of evidence synthesis, increased potential ROB, raised applicability concerns, and should be avoided in future manuscripts. In addition to that, some studies have been based on small groups of patients [27, 33, 36, 37]. Thus, although their reported accuracy may be high, such models tend to overfit data owing too small sample sizes which reduces its generalization capabilities and their potential applicability in clinical setting [19]. Almost all algorithms were evaluated using internal validation methods, most commonly with the use of the tenfold or fivefold cross-validation protocol. Only four studies used additional internal hold-out test dataset [4, 31, 32, 40]. In addition to that, only one AI algorithm was validated externally [42], which raises concerns about the potential validity of AI in real-world clinical environments [19].

Moreover, due to large variety of the studied cohorts (mean age, share of male sex, number of included patients), as well as due to the aforementioned incompleteness of information regarding the investigated cohorts, it was not possible to perform a robust meta-analysis of the reviewed studies. Furthermore, reporting of data on multiple outcomes with different measures and follow-up durations makes direct comparison of the studies problematic, creates opportunities for “cherry-picking” of the primary endpoints, and reduces applicability of the results in clinical practice [56].

In addition to that, sometimes only scarce data on the prediction quality were available (AUC or accuracy without corresponding sensitivity and specificity of the AI model). Thus, some studies were analyzed based on just one of the metrics and direct comparisons of the results were not possible. Moreover, accuracy and reliability of machine learning models are highly dependent on the quality as well as the completeness of both the training and validation data used [57]. To directly compare the performance of different AI models as well as to compare their performance with the accuracy of recommendation-based CRT response rates, one would need to train and evaluate them on exactly the same data.

Finally, many algorithms, especially the state-of-the-art deep learning models, are “black box algorithms” and the explainability of their prediction mechanisms remains unknown. As the determination of the output by the model is not transparent, it remains under debate if such algorithms should be implemented in the daily clinical practice [19].

### Clinical implications

Unsupervised AI models were able to identify clusters of patients with significantly different rates of primary outcome events (death, heart failure event). In comparison to the guideline-based CRT response prediction accuracy of 70%, supervised AI models trained on cohorts with > 100 patients achieved up to 85% accuracy and AUC of 0.86 of CRT response prediction for echocardiographic and clinical outcomes, respectively. Thus, AI models seem to be a valuable tool in phenotyping of patients eligible for CRT implantation and predicting potential responders. However, these findings must be validated in RCTs. In addition to that, AI algorithms demonstrated that a holistic approach to the problem of CRT responder selection must be adopted, including evaluation of a wide range of cardiovascular disease risk factors. These findings must also be validated in RCTs. Moreover, forthcoming studies should be reported more thoroughly, to make it possible to perform more robust literature synthesis and meta-analysis.

## Conclusion

AI models seem to be an accurate and clinically applicable tool in phenotyping of patients eligible for CRT implantation and predicting potential responders (Fig. 4). Synergy of machine-aided data analysis and human-driven interpretation of the results seems to be possible. In the future, AI may help to increase CRT response rates to over 80% and improve clinical decision-making and prognosis of the patients, including reduction of mortality rates. However, these hypotheses must be validated in randomized controlled trials.

### Supplementary Information

Below is the link to the electronic supplementary material.

Supplementary file1 (DOCX 29 KB)

Supplementary file2 (XLSX 28 KB)

## Data Availability

All additional data are available in the Supplementary Material S1 and Supplementary Tables S2, S3, and S4.
