# Supplementary material for: Artificial intelligence models in prediction of response to cardiac resynchronization therapy: a systematic review
Source: Heart Fail Rev. 2023 Oct 20;29(1):133–50. doi: 10.1007/s10741-023-10357-8 (PMC10904439; doi:10.1007/s10741-023-10357-8)
Supplement: Supplementary file 1 — Supplementary file1 (DOCX 29 KB) [file 10741_2023_10357_MOESM1_ESM.docx]

**Full search strategy for each database**

PubMed:174 results

Search query:

("cardiac resynchronization therapy"[MeSH Terms] OR ("cardiac"[All Fields] AND "resynchronization"[All Fields] AND "therapy"[All Fields]) OR "cardiac resynchronization therapy"[All Fields] OR ("cardiac"[All Fields] AND "resynchronisation"[All Fields] AND "therapy"[All Fields]) OR "cardiac resynchronisation therapy"[All Fields] OR ("dyssynchronies"[All Fields] OR "dyssynchrony"[All Fields])) AND ("electrocardiography"[MeSH Terms] OR "electrocardiography"[All Fields] OR "ecg"[All Fields] OR ("electrocardiographies"[All Fields] OR "electrocardiography"[MeSH Terms] OR "electrocardiography"[All Fields]) OR ("electrocardiogram s"[All Fields] OR "electrocardiography"[MeSH Terms] OR "electrocardiography"[All Fields] OR "electrocardiogram"[All Fields] OR "electrocardiograms"[All Fields]) OR ("echocardiographies"[All Fields] OR "echocardiography"[MeSH Terms] OR "echocardiography"[All Fields]) OR (("cardiacs"[All Fields] OR "heart"[MeSH Terms] OR "heart"[All Fields] OR "cardiac"[All Fields]) AND ("diagnostic imaging"[MeSH Subheading] OR ("diagnostic"[All Fields] AND "imaging"[All Fields]) OR "diagnostic imaging"[All Fields] OR "ultrasound"[All Fields] OR "ultrasonography"[MeSH Terms] OR "ultrasonography"[All Fields] OR "ultrasonics"[MeSH Terms] OR "ultrasonics"[All Fields] OR "ultrasounds"[All Fields] OR "ultrasound s"[All Fields])) OR ("echocardiography"[MeSH Terms] OR "echocardiography"[All Fields] OR "echocardiogram"[All Fields] OR "echocardiograms"[All Fields]) OR ("tomography, x ray computed"[MeSH Terms] OR ("tomography"[All Fields] AND "x ray"[All Fields] AND "computed"[All Fields]) OR "x-ray computed tomography"[All Fields] OR ("computed"[All Fields] AND "tomography"[All Fields]) OR "computed tomography"[All Fields]) OR ("j comput tomogr"[Journal] OR "commun theory"[Journal] OR "child teenagers"[Journal] OR "cancer ther"[Journal] OR "ct"[All Fields]) OR (("magnet s"[All Fields] OR "magnetical"[All Fields] OR "magnetically"[All Fields] OR "magnetics"[MeSH Terms] OR "magnetics"[All Fields] OR "magnetic"[All Fields] OR "magnetisation"[All Fields] OR "magnetisations"[All Fields] OR "magnetised"[All Fields] OR "magnetism"[All Fields] OR "magnetisms"[All Fields] OR "magnetization"[All Fields] OR "magnetizations"[All Fields] OR "magnetize"[All Fields] OR "magnetized"[All Fields] OR "magnetizing"[All Fields] OR "magnets"[MeSH Terms] OR "magnets"[All Fields] OR "magnet"[All Fields]) AND "resonanse"[All Fields] AND ("image"[All Fields] OR "image s"[All Fields] OR "imaged"[All Fields] OR "imager"[All Fields] OR "imager s"[All Fields] OR "imagers"[All Fields] OR "images"[All Fields] OR "imaging"[All Fields] OR "imaging s"[All Fields] OR "imagings"[All Fields])) OR ("magnetic resonance imaging"[MeSH Terms] OR ("magnetic"[All Fields] AND "resonance"[All Fields] AND "imaging"[All Fields]) OR "magnetic resonance imaging"[All Fields] OR "mri"[All Fields]) OR ("tomography, emission computed, single photon"[MeSH Terms] OR ("tomography"[All Fields] AND "emission computed"[All Fields] AND "single photon"[All Fields]) OR "single-photon emission-computed tomography"[All Fields] OR ("single"[All Fields] AND "photon"[All Fields] AND "emission"[All Fields] AND "computed"[All Fields] AND "tomography"[All Fields]) OR "single photon emission computed tomography"[All Fields]) OR ("spect s"[All Fields] OR "tomography, emission computed, single photon"[MeSH Terms] OR ("tomography"[All Fields] AND "emission computed"[All Fields] AND "single photon"[All Fields]) OR "single-photon emission-computed tomography"[All Fields] OR "spect"[All Fields] OR "spects"[All Fields])) AND ("machine learning"[MeSH Terms] OR ("machine"[All Fields] AND "learning"[All Fields]) OR "machine learning"[All Fields] OR ("antagonists and inhibitors"[MeSH Subheading] OR ("antagonists"[All Fields] AND "inhibitors"[All Fields]) OR "antagonists and inhibitors"[All Fields] OR "ai"[All Fields]) OR ("artificial intelligence"[MeSH Terms] OR ("artificial"[All Fields] AND "intelligence"[All Fields]) OR "artificial intelligence"[All Fields]) OR ("artificial intelligence"[MeSH Terms] OR ("artificial"[All Fields] AND "intelligence"[All Fields]) OR "artificial intelligence"[All Fields] OR ("machine"[All Fields] AND "intelligence"[All Fields]) OR "machine intelligence"[All Fields]) OR "k-means"[All Fields] OR ("random forest"[MeSH Terms] OR ("random"[All Fields] AND "forest"[All Fields]) OR "random forest"[All Fields]) OR (("gradient"[All Fields] OR "gradient s"[All Fields] OR "gradients"[All Fields]) AND ("boost"[All Fields] OR "boosted"[All Fields] OR "boosting"[All Fields] OR "boosts"[All Fields])) OR ("support vector machine"[MeSH Terms] OR ("support"[All Fields] AND "vector"[All Fields] AND "machine"[All Fields]) OR "support vector machine"[All Fields] OR ("support"[All Fields] AND "vector"[All Fields] AND "machines"[All Fields]) OR "support vector machines"[All Fields]) OR ("decision trees"[MeSH Terms] OR ("decision"[All Fields] AND "trees"[All Fields]) OR "decision trees"[All Fields] OR ("decision"[All Fields] AND "tree"[All Fields]) OR "decision tree"[All Fields]) OR "lstm"[All Fields] OR ("long"[All Fields] AND ("short"[All Fields] OR "shorts"[All Fields]) AND ("term birth"[MeSH Terms] OR ("term"[All Fields] AND "birth"[All Fields]) OR "term birth"[All Fields] OR "term"[All Fields]) AND ("network"[All Fields] OR "network s"[All Fields] OR "networked"[All Fields] OR "networker"[All Fields] OR "networkers"[All Fields] OR "networking"[All Fields] OR "networks"[All Fields])) OR ("encodable"[All Fields] OR "encode"[All Fields] OR "encoded"[All Fields] OR "encoder"[All Fields] OR "encoders"[All Fields] OR "encodes"[All Fields] OR "encoding"[All Fields] OR "encodings"[All Fields]) OR ("decodable"[All Fields] OR "decode"[All Fields] OR "decoded"[All Fields] OR "decoder"[All Fields] OR "decoder s"[All Fields] OR "decoders"[All Fields] OR "decodes"[All Fields] OR "decoding"[All Fields]) OR "tensorflow"[All Fields] OR "pytorch"[All Fields] OR "keras"[All Fields] OR (("classification"[MeSH Terms] OR "classification"[All Fields] OR "classifications"[All Fields] OR "classification"[MeSH Subheading] OR "classification s"[All Fields] OR "classificator"[All Fields] OR "classificators"[All Fields]) AND ("algorithm s"[All Fields] OR "algorithmic"[All Fields] OR "algorithmically"[All Fields] OR "algorithmics"[All Fields] OR "algorithmization"[All Fields] OR "algorithms"[MeSH Terms] OR "algorithms"[All Fields] OR "algorithm"[All Fields])) OR (("organization and administration"[MeSH Terms] OR ("organization"[All Fields] AND "administration"[All Fields]) OR "organization and administration"[All Fields] OR "supervision"[All Fields] OR "supervise"[All Fields] OR "supervised"[All Fields] OR "supervises"[All Fields] OR "supervising"[All Fields] OR "supervisions"[All Fields]) AND ("learning"[MeSH Terms] OR "learning"[All Fields] OR "learn"[All Fields] OR "learned"[All Fields] OR "learning s"[All Fields] OR "learnings"[All Fields] OR "learns"[All Fields])) OR ("unsupervised"[All Fields] AND ("learning"[MeSH Terms] OR "learning"[All Fields] OR "learn"[All Fields] OR "learned"[All Fields] OR "learning s"[All Fields] OR "learnings"[All Fields] OR "learns"[All Fields])) OR ("cluster analysis"[MeSH Terms] OR ("cluster"[All Fields] AND "analysis"[All Fields]) OR "cluster analysis"[All Fields] OR "clustering"[All Fields] OR "clusterings"[All Fields] OR "cluster"[All Fields] OR "cluster s"[All Fields] OR "clustered"[All Fields] OR "clusterization"[All Fields] OR "clusters"[All Fields]) OR ("deep learning"[MeSH Terms] OR ("deep"[All Fields] AND "learning"[All Fields]) OR "deep learning"[All Fields]) OR ("deep"[All Fields] AND ("neural networks, computer"[MeSH Terms] OR ("neural"[All Fields] AND "networks"[All Fields] AND "computer"[All Fields]) OR "computer neural networks"[All Fields] OR ("neural"[All Fields] AND "network"[All Fields]) OR "neural network"[All Fields])) OR "cnn"[All Fields] OR (("convolute"[All Fields] OR "convoluted"[All Fields] OR "convolutes"[All Fields] OR "convoluting"[All Fields] OR "convolution"[All Fields] OR "convolutional"[All Fields] OR "convolutions"[All Fields] OR "convolutive"[All Fields]) AND ("neural networks, computer"[MeSH Terms] OR ("neural"[All Fields] AND "networks"[All Fields] AND "computer"[All Fields]) OR "computer neural networks"[All Fields] OR ("neural"[All Fields] AND "network"[All Fields]) OR "neural network"[All Fields])) OR ("int j comput vis"[Journal] OR ("computer"[All Fields] AND "vision"[All Fields]) OR "computer vision"[All Fields]) OR "rnn"[All Fields] OR (("recurrance"[All Fields] OR "recurrence"[MeSH Terms] OR "recurrence"[All Fields] OR "recurrences"[All Fields] OR "recurrencies"[All Fields] OR "recurrency"[All Fields] OR "recurrent"[All Fields] OR "recurrently"[All Fields] OR "recurrents"[All Fields]) AND ("neural networks, computer"[MeSH Terms] OR ("neural"[All Fields] AND "networks"[All Fields] AND "computer"[All Fields]) OR "computer neural networks"[All Fields] OR ("neural"[All Fields] AND "network"[All Fields]) OR "neural network"[All Fields])))

Scopus: 182 results

Search query:

(TITLE-ABS-KEY("cardiac resynchronisation therapy" OR "dyssynchrony") AND TITLE-ABS-KEY("ecg" OR "electrocardiography" OR "electrocardiogram" OR "echocardiography" OR "cardiac ultrasound" OR "echocardiogram" OR "computed tomography" OR "ct" OR "magnetic resonanse imaging" OR "mri" OR "Single-photon emission computed tomography" OR "spect") AND TITLE-ABS-KEY("machine learning" OR "ai" OR "artificial intelligence" OR "machine intelligence" OR "k-means" OR "random forest" OR "gradient boost" OR "support vector machines" OR "decision tree" OR "lstm" OR "long short term network" OR "encoder" OR "decoder" OR "tensorflow" OR "pytorch" OR "keras" OR "classification algorithm" OR "supervised learning" OR "unsupervised learning" OR "clustering" OR "deep learning" OR "deep neural network" OR "cnn" OR "convolutional neural network" OR "computer vision" OR "rnn" OR "recurrent neural network"))

Embase: 297 results

Search query:

('cardiac resynchronisation therapy'/exp OR 'cardiac resynchronisation therapy' OR (('cardiac'/exp OR cardiac) AND resynchronisation AND ('therapy'/exp OR therapy)) OR 'dyssynchrony'/exp OR dyssynchrony) AND ('ecg'/exp OR ecg OR 'electrocardiography'/exp OR electrocardiography OR 'electrocardiogram'/exp OR electrocardiogram OR 'echocardiography'/exp OR echocardiography OR 'cardiac ultrasound' OR (('cardiac'/exp OR cardiac) AND ('ultrasound'/exp OR ultrasound)) OR 'echocardiogram'/exp OR echocardiogram OR 'computed tomography'/exp OR 'computed tomography' OR (computed AND ('tomography'/exp OR tomography)) OR 'ct'/exp OR ct OR 'magnetic resonanse imaging' OR (magnetic AND resonanse AND ('imaging'/exp OR imaging)) OR 'mri'/exp OR mri OR 'single-photon emission computed tomography'/exp OR 'single-photon emission computed tomography' OR ('single photon' AND ('emission'/exp OR emission) AND computed AND ('tomography'/exp OR tomography)) OR 'spect'/exp OR spect) AND ('machine learning'/exp OR 'machine learning' OR (('machine'/exp OR machine) AND ('learning'/exp OR learning)) OR ai OR 'artificial intelligence'/exp OR 'artificial intelligence' OR (artificial AND ('intelligence'/exp OR intelligence)) OR 'machine intelligence'/exp OR 'machine intelligence' OR (('machine'/exp OR machine) AND ('intelligence'/exp OR intelligence)) OR 'k means'/exp OR 'k means' OR 'random forest'/exp OR 'random forest' OR (random AND ('forest'/exp OR forest)) OR 'gradient boost' OR (('gradient'/exp OR gradient) AND ('boost'/exp OR boost)) OR 'support vector machines'/exp OR 'support vector machines' OR (('support'/exp OR support) AND ('vector'/exp OR vector) AND machines) OR 'decision tree'/exp OR 'decision tree' OR (('decision'/exp OR decision) AND ('tree'/exp OR tree)) OR lstm OR 'long short term network' OR (long AND short AND term AND ('network'/exp OR network)) OR 'encoder'/exp OR encoder OR decoder OR 'tensorflow'/exp OR tensorflow OR 'pytorch'/exp OR pytorch OR keras OR 'classification algorithm'/exp OR 'classification algorithm' OR (('classification'/exp OR classification) AND ('algorithm'/exp OR algorithm)) OR 'supervised learning'/exp OR 'supervised learning' OR (supervised AND ('learning'/exp OR learning)) OR 'unsupervised learning'/exp OR 'unsupervised learning' OR (unsupervised AND ('learning'/exp OR learning)) OR 'clustering'/exp OR clustering OR 'deep learning'/exp OR 'deep learning' OR (deep AND ('learning'/exp OR learning)) OR 'deep neural network'/exp OR 'deep neural network' OR (deep AND neural AND ('network'/exp OR network)) OR cnn OR 'convolutional neural network'/exp OR 'convolutional neural network' OR (convolutional AND neural AND ('network'/exp OR network)) OR 'computer vision'/exp OR 'computer vision' OR (('computer'/exp OR computer) AND ('vision'/exp OR vision)) OR rnn OR 'recurrent neural network'/exp OR 'recurrent neural network' OR (recurrent AND neural AND ('network'/exp OR network)))

Cochrane: 12 results

Search query:

(cardiac resynchronisation therapy OR dyssynchrony) AND (ecg OR electrocardiography OR electrocardiogram OR echocardiography OR cardiac ultrasound OR echocardiogram OR computed tomography OR ct OR magnetic resonanse imaging OR mri OR Single-photon emission computed tomography OR spect) AND (machine learning OR ai OR artificial intelligence OR machine intelligence OR k-means OR random forest OR gradient boost OR support vector machines OR decision tree OR lstm OR long short term network OR encoder OR decoder OR tensorflow OR pytorch OR keras OR classification algorithm OR supervised learning OR unsupervised learning OR clustering OR deep learning OR deep neural network OR cnn OR convolutional neural network OR computer vision OR rnn OR recurrent neural network) in Title Abstract Keyword - (Word variations have been searched)

**PROBAST assessment**

| **Author and date** | **ROB participants** | **ROB predictors** | **ROB outcome** | **ROB analysis** | **Applicability participants** | **Applicability predictors** | **Applicability outcome** | **Overall ROB** | **Overall applicability** |
| --- | --- | --- | --- | --- | --- | --- | --- | --- | --- |
| Bivona D. et al. (2022) | 1 | 1 | 1 | 1 | 1 | 1 | 1 | 1 | 1 |
| Cai C. et al. (2021) | ? | 1 | 1 | 1 | ? | 1 | 1 | ? | ? |
| Feeny A. et al. (2019) | ? | 1 | 1 | 1 | ? | 1 | 1 | ? | ? |
| Fernandes et al. (2023) | 1 | 1 | 1 | 1 | 1 | 1 | 1 | 1 | 1 |
| Field M. et al. (2020) | 1 | 1 | 1 | 1 | 1 | 1 | 1 | 1 | 1 |
| Gallard A. et al. (2020) | 1 | 1 | 1 | ? | 1 | 1 | 1 | ? | 1 |
| Galli E. et al. (2021) | 1 | 1 | 1 | 1 | 1 | 1 | 1 | 1 | 1 |
| Haque et al. (2022) | 1 | 1 | 1 | 1 | 1 | 1 | 1 | 1 | 1 |
| HE et al. (2023) | 1 | 1 | 1 | 1 | 1 | 1 | 1 | 1 | 1 |
| Hong et al. (2022) | 1 | 1 | 1 | 1 | 1 | 1 | 1 | 1 | 1 |
| Howell S. et al. (2021) | 1 | 1 | 1 | 1 | 1 | 1 | 1 | 1 | 1 |
| Hu S. et al. (2019) | ? | 1 | 1 | ? | ? | 1 | 1 | ? | ? |
| Kalscheur M. et al. (2018) | 1 | 1 | 1 | ? | 1 | 1 | 1 | ? | 1 |
| Lei J. et al. (2019) | 1 | 1 | 1 | 1 | 1 | 1 | 1 | 1 | 1 |
| Liang Y. et al. (2021) | ? | 1 | 1 | 1 | ? | 1 | 1 | ? | ? |
| Schmitz B. et al. (2014) | 1 | 1 | 1 | 1 | 1 | 1 | 1 | 1 | 1 |
| Tokodi M. et al. (2020) | ? | 1 | 1 | 1 | ? | 1 | 1 | ? | ? |
| Wouters et al. (2023) | 1 | 1 | 1 | 1 | 1 | 1 | 1 | 1 | 1 |
